# Prepubertal castration eliminates sex differences in lifespan and growth trajectories in genetically heterogeneous mice

**DOI:** 10.1111/acel.13891

**Published:** 2023-05-23

**Authors:** Nisi Jiang, Catherine J. Cheng, Jonathan Gelfond, Randy Strong, Vivian Diaz, James F. Nelson

**Affiliations:** ^1^ The Sam and Ann Barshop Institute for Longevity and Aging Studies, UT Health San Antonio San Antonio Texas USA; ^2^ Department of Cellular and Integrative Physiology UT Health San Antonio San Antonio Texas USA; ^3^ Department of Population Health Sciences UT Health San Antonio San Antonio Texas USA; ^4^ Department of Pharmacology UT Health San Antonio San Antonio Texas USA; ^5^ Research Service of the South Texas Veterans Health Care System San Antonio Texas USA

**Keywords:** age‐specific mortality, aging, body composition, body weight, castration, growth, lifespan, sex differences

## Abstract

Sex differences in aging and longevity have been widely observed, with females consistently outliving males across human populations. However, the mechanisms driving these disparities remain poorly understood. In this study, we explored the influence of post‐pubertal testicular effects on sex differences in aging by prepubertally castrating genetically heterogeneous (UM‐HET3) mice, a unique mouse model that emulates human sex differences in age‐related mortality. Prepubertal castration eliminated the longevity disparity between sexes by reducing the elevated early‐ to mid‐life mortality rate observed in males and extending their median lifespan to match that of females. Additionally, castration extended the duration of body weight growth and attenuated the inverse correlation between early‐age body weight and lifespan in males, aligning their growth trajectories with those of females. Our findings suggest that post‐pubertal testicular actions in genetically diverse mice are primarily responsible for sex differences in longevity as well as growth trajectories. These findings offer a foundation for further investigation into the fundamental mechanisms driving sex‐specific aging patterns and the development of potential pro‐longevity interventions.

## Graphical abstract

Males live shorter than females and experience higher mortality rates since early adulthood with no known definitive mechanisms. Here we found prepubertal castration eliminated sex differences in longevity and growth in a genetically heterogeneous mouse model, which indicates the presence of testes plays a causative role in these sex differences.

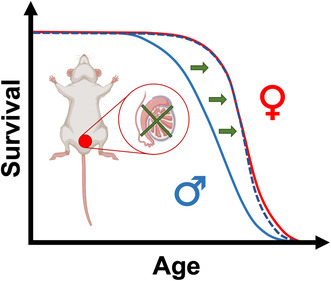

**Abbreviations**
CR: calorie restriction
IGF‐1: insulin‐like growth factor 1
ITP: Intervention Testing Program
NIA: National Institute on Aging
ORX: orchiectomized
SEM: standard error of the mean
SHAM: Sham‐operated

Human males live shorter and experience higher mortality rates than females throughout the lifespan, with the greatest difference during early adulthood (Austad & Fischer, 2016; Gems, 2014). However, elucidating the mechanisms that underlie this sex difference has been impeded by the absence of mouse models with consistent sex differences in lifespan (Austad & Fischer, 2016; Cheng et al., 2019). Genetically heterogeneous UM‐HET3 mice, used in the NIA Interventions Testing Program (ITP) for over 15 years, have a robust, reproducible sex difference in longevity like that of humans (Cheng et al., 2019). Male UM‐HET3 mice have an elevated mortality hazard compared to females in early adulthood that steadily diminishes thereafter, and thus are a unique model to investigate the mechanisms underlying sex differences in aging and longevity.

In mammals, the gonads account for many sex differences, although genes on the X and Y chromosomes other than those controlling gonadal differentiation also play a role (Arnold & Chen, 2009; Davis et al., 2019). Castration is reported to prolong lifespan and slow epigenetic aging in males among several species (Min et al., 2012; Sugrue et al., 2021), whereas ovariectomy has been shown to have mixed effects on female longevity (Benedusi et al., 2015; Cargill et al., 2003). These findings suggest that androgens or other testicular products shorten life, leading to the sex difference in longevity. However, the absence until recently of a mouse model for human sex differences in aging and underpowered experimental designs prevented addressing if the testes affect sex differences (1) in mortality across the entire lifespan or only during a portion of it, (2) in growth rates and body composition during aging, and (3) in the inverse relationship between body weight and longevity. This study is the first with the power to address these questions in a mouse model of human sex differences in longevity.

Males are exposed to elevated testicular steroids at three distinct periods of life: first during early fetal development (O'Shaughnessy et al., 2006); second, during a brief period shortly after birth (Clarkson & Herbison, 2016); and finally, from puberty onward, declining gradually with advancing age (Jean‐Faucher et al., 1978). These three periods play important roles in sexual development, but the importance of these three periods of hormone exposure to aging processes is largely unknown.

We directly tested the role of the post‐pubertal gonadal function in sex differences in aging by castrating mice just before the onset of puberty. Mice were bilaterally orchiectomized (ORX, *n* = 238) or sham‐operated (SHAM, *n* = 238) prepubertally, before postnatal day 30. Median lifespan increased from 771 days in the SHAM controls to 850 days in ORX mice (*p* = 0.0123) (Figure 1a). The large sample size of this study provided power to assess the effects of castration on the early‐to‐midlife male‐specific elevation in the mortality hazard of UM‐HET3 male mice (Cheng et al., 2019). Prepubertal castration eliminated this elevation but, notably, had no effect on the mortality hazard after midlife when the sexes do not differ in mortality rate (Figure 1b). Although there were no unoperated males or females in this study, survival data obtained in our colony from 12 separate cohorts of untreated males and females since 2004 provides a basis for comparison (Figure 1c,d). Median lifespans and Kaplan–Meier survival of historical males and females are indistinguishable from those of the sham‐operated and castrated males, respectively (Figures 1e, and S1). Thus, prepubertal castration reduced the age‐specific mortality hazard of males to a level indistinguishable from that of the historical females (Figure 1f). In males, the testes thus drive the sex differences in age‐specific mortality and longevity, but they have limited influence on survival in aging mice after midlife.

**FIGURE 1:**
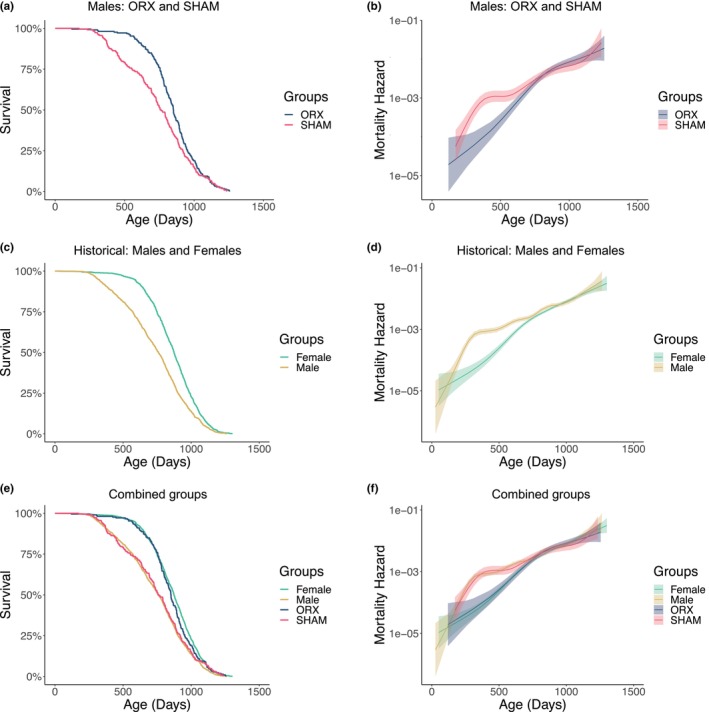
Prepubertal castration prolonged lifespan and eliminated sex differences in the age‐specific mortality hazard. (a) Survival curves (SHAM, *n* = 231; ORX, *n* = 221; median lifespan SHAM vs. ORX, 771 vs. 850 days; Log‐rank test, *p* = 0.0123) and (b) age‐specific mortality hazard rate for ORX or SHAM males. (c) Survival curves (Male, *n* = 1281; Female, *n* = 1017; median lifespan Male vs. Female, 760 vs. 874 days; Log‐rank test, *p* < 0.0001) and (d) age‐specific mortality hazard rate for historical ITP control males and females. (e) Combined survival curves (Log‐rank test, Males vs. SHAM, *p* = 0.2877; Female vs. ORX, *p* = 0.1117). (f) Age‐specific mortality hazard rate for all groups.

We previously reported that male UM‐HET3 mice weigh significantly more than females across the lifespan (Cheng et al., 2019). Higher body weight is correlated with decreased lifespan within species (Miller et al., 2002). We, therefore, asked whether the testes play a role in the sex differences in body weight and growth, which may contribute to the shorter lifespan of males. Prepubertal castration significantly reduced the growth rate between 1 and 6 months (Figure 2a), mainly by slowing the accumulation of lean body mass (Figure 2b). Since the major bodyweight difference occurred between weaning and 6 months, we measured IGF‐1 levels at 4 months but found no significant effect of castration (Figure 2c), suggesting that castration slows growth by other mechanisms.

**FIGURE 2:**
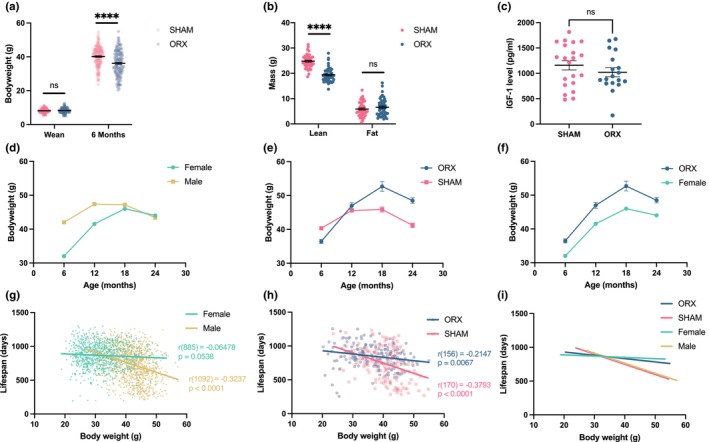
Prepubertal castration shifted growth pattern and correlation between bodyweight and lifespan to female levels. (a) Body weights at wean and 6 months (Wean SAHM, *n* = 222; Wean ORX, *n* = 207; 6 months SHAM, *n* = 172; 6 months ORX, *n* = 158; *t*‐test, wean, *p* = 0.2713; 6 months, *p* < 0.0001). (b) Body composition at 4 months (SHAM, *n* = 42; ORX, *n* = 45; *t*‐test, Lean mass, *p* < 0.0001; Fat mass, *p* = 0.2975). (c) Plasma IGF‐1 levels at 4 months (SHAM, *n* = 21; ORX, *n* = 18; *t*‐test, *p* = 0.2941). (d) Bodyweight changes in historical ITP control mice, (e) castrated and sham‐operated male mice, and (f) combined historical ITP female and castrated male groups. (g) The correlation between bodyweight at 6 months to lifespan in historical ITP control mice months (Male, *n* = 1094; Female, *n* = 887), (h) castrated and sham‐operated male mice, (SHAM, *n* = 172; ORX, *n* = 158), and (i) combined groups. All bars and error bars represent mean ± SEM.

Castration also eliminated the sex difference in the duration of growth. UM‐HET3 males stop growing around 12 months, while females continue growing until 18 months (Figure 2d, historical data). Although castration reduced body weight at 6 months, by 12 months body weight of castrated mice caught up with and by 18 months exceeded that of sham‐operated controls, which extended the duration of growth by around 6 months (Figure 2e). As a result, castration shifted the duration of growth and subsequent decline from the male pattern to that of females (Figure 2f). Castration also shifted the stronger inverse relationship between body weight at 6 months and lifespan in males to the weaker one in females (Figure 2g–i). Thus, prepubertal castration shifted both the shape of the growth curve and the correlation between body weight and lifespan to that of females.

Although previous studies have shown that castration can extend the average lifespan, this is the first to show that post‐pubertal gonadal hormones or other factors are responsible for the selectively elevated mortality hazard of males from puberty to midlife. In addition, the results indicate that the testes underlie sex differences in growth duration and the correlation between body weight and lifespan. The stronger negative correlation between early‐age body weight and lifespan in males compared to females reported previously indicates greater sensitivity or less resilience of males to life‐shortening factors associated with elevated body weight (Bou Sleiman et al., 2022). That castration weakened this negative correlation indicates a primary role of the testes in the greater sensitivity of males to the lifespan‐shortening effects of higher body weight.

The sex‐specific elevation of mortality in human males during early adulthood has been hypothesized to reflect androgen‐driven increases in risk‐taking behaviors (Carnes & Olshansky, 1997). However, in laboratory‐housed mice, risk‐taking behaviors are limited to fighting. To minimize the effects of fighting on mortality, all mice in cages with overt fighting (see methods) were censored from this study and the historical studies referenced here. However, mice with minor wounds were not censored. Indeed, fewer cases of minor wounding were observed in castrated mice (3 of 238 mice) than in sham‐operated mice (7 of 238 mice). We questioned if mice with minor wounds lived shorter. Surprisingly, mice from cages with minor wounding tended to live longer than those without wounding (Figure S2). Although reductions in fighting do not appear to play a major role in the reduced mortality of castrated mice, we cannot exclude a role for a reduction in more nuanced testis‐driven social hierarchy and stress, since subordination in male mice can lead to inflammation and cardio‐pathology (Razzoli et al., 2018). Pathological assessment of mortality of intact and castrated males is needed to gain further insight into the basis for and the mechanisms underlying increased mortality of intact males.

UM‐HET3 males have a shorter duration of growth than females in all cohorts from ITP. Prepubertal castration prolonged both duration of growth and lifespan, which provides one more example of a life‐extending intervention that prolongs growth, as previously shown in calorie restriction (CR) (McCay et al., 1935) and growth hormone/IGF‐1 deficiencies (Bartke et al., 2001; Sun et al., 2017). The hypothesis that the duration of growth may limit lifespan is not new. Since McCay first proposed using CR to preserve the “growth power” to prolong the lifespan, researchers have posited that developmental processes involved in growth forestall or override the processes that lead to senescence (McCay et al., 1935; Sun et al., 2017). Castration differs from the other life‐extending interventions that prolong the duration of growth in that it increases body weight whereas CR and growth hormone/IGF‐1 deficiencies reduce body weight, thereby strengthening the role of growth duration over body weight as a determinant of longevity.

Our results suggest that post‐pubertal exposure to androgens plays a critical role in causing the sex differences in aging reported here, although we cannot rule out a role for other testicular hormones. Attention can next focus on whether exposure to testicular hormones throughout post‐pubertal life or only during a segment of adulthood, such as the immediate peri‐pubertal period, is responsible for these sex differences in aging, and the underlying mechanisms. Identifying the mechanisms underlying these sex differences is important for developing therapeutic targets that could abrogate the deleterious effects of androgens on aging yet maintain male sexual function. Notably, the Interventions Testing Program has identified several drugs that increase lifespan only in male UM‐HET3 mice (Austad & Fischer, 2016). Research to determine if any of those compounds do so without compromising male reproductive function is needed.

## AUTHOR CONTRIBUTIONS

Conceptualization: CJC, NJ, JN, RS. Methodology: CJC, NJ, JN, RS, JG. Investigation: NJ, CJC, VD. Funding acquisition: RS, JN. Data analysis: NJ, CJC, JG, JN. Writing: NJ, CJC, JN.

## FUNDING INFORMATION

National Institute on Aging grant 5U01AG022307 (RS, JN). National Institute on Aging grant 5P30AG013319 (RS, JN).

## CONFLICT OF INTEREST STATEMENT

The authors declare no conflict of interest.

## Supporting information


Figure S1

## Data Availability

The data that support the findings of this study are available from the corresponding author upon reasonable request.
