# Supplementary material for: Prepubertal castration eliminates sex differences in lifespan and growth trajectories in genetically heterogeneous mice
Source: Aging Cell. 2023 May 23;22(8):e13891. doi: 10.1111/acel.13891 (PMC10410013; doi:10.1111/acel.13891)
Supplement: Supplementary file 1 — Figure S1 [file ACEL-22-e13891-s001.docx]

Materials and Methods

Animal

All mouse studies were conducted in compliance with Institutional Animal Care and Use Committee guidelines (IACUC 20040042AR). All animals are bred and kept following the same protocol as ITP and housed within the same room as ITP mice to keep the comparability. Genetically heterogeneous UM-HET3 mice were bred from a standardized four-way cross between BALB/cByJ x C57BL/6J F1 mothers and C3H/HeJ x DBA/2J F1 fathers as previously described(Miller et al., 2007). 19- to 21-day-old weanlings were collected and equally assigned into either ORX or SHAM groups. Mice were maintained following animal husbandry protocols used by the National Institute on Aging Interventions Testing Program(Miller et al., 2007). In brief, mice were housed under 25 °C, 12/12 h light/dark cycle with free access to Purina 5LG6 food. Three males or Five females were housed per cage. Male mice for castration and sham-operation were collected at the same time, while the non-surgical control mice data are extracted from Intervention testing program database.

Body weight and composition

Mice were weighed every 2 weeks until the age of 6 months. Follow-up weights were collected at 12, 18, and 24 months of age. The body composition of the mice was evaluated at 4 months by quantitative magnetic resonance imaging with an EchoMRI body composition analyzer (Echo Medical Systems, Houston, Texas).

Survival protocol

Mice were examined at least daily for signs of illness. If mice were considered unlikely to survive for more than an additional 48 hours by an experienced technician, including but not limited to severely ulcerated or bleeding tumors and inability to eat or drink, they were euthanized for humane reasons (ORX n = 8, SHAM n = 5). For moribund mice, the age of euthanasia was recorded as the best available estimate of the natural lifespan. For mice that were found dead during the daily inspection, the age at inspection was recorded as the lifespan. Bodies were fixed for later necropsy analysis.

Censoring and euthanasia for fight wounds

Mice were euthanized if fighting resulted in severe wounds (bleeding/infected wounds or wounds found on more than 20% of the body surface area) on at least one mouse within a cage. Once the decision to euthanize was confirmed, all mice in the cage were euthanized.

Removal of mice from the longevity population

Mice (n = 24) terminated because of fighting (SHAM n = 3), cage flooding (ORX n = 8), and experimental procedure (SHAM n = 4, ORX n = 9) were removed from this study.

Surgical Castration

Male UM-HET3 mice in the castration group (n=238) received bilateral orchidectomy via midline scrotal incision under inhalant isoflurane (3-4% isoflurane in a 100% oxygen mix) anesthesia within 30 days after birth, as previously described(Rettew, Huet-Hudson, & Marriott, 2008). Another group of male mice were kept intact (n=238) and received a sham operation. The sham operation consisted of a midline scrotal incision and manipulation of the testicular fat pad without removal. All mice that died within 2 weeks post-surgery were considered surgery-related death and removed from the study.

Serum IGF-1 level measurement

Plasma samples (20 μL) were collected by tail venipuncture at 16 weeks of age. Plasma IGF-1 levels were quantified via an enzyme-linked immunosorbent assay using the Mouse/Rat IGF-I Quantikine ELISA Kit (R&D Systems, Minneapolis, MN) according to the manufacturer’s instructions.

Statistical Analysis

All statistical analysis were conducted in GraphPad Prism. Survival data were compared using Kaplan-Meier (log rank) test, all censored mice are removed from survival analysis and the calculation of median lifespan. Body weight, body composition, and plasma IGF-1 level data were compared using t-test. Linear regressions were conducted to estimate correlations between bodyweight and lifespan. Age-specific mortality rates were calculated by using R package *dplyr* and *bshazard* under the R environment for statistical computing 3.5.1.

**Supplementary Materials**


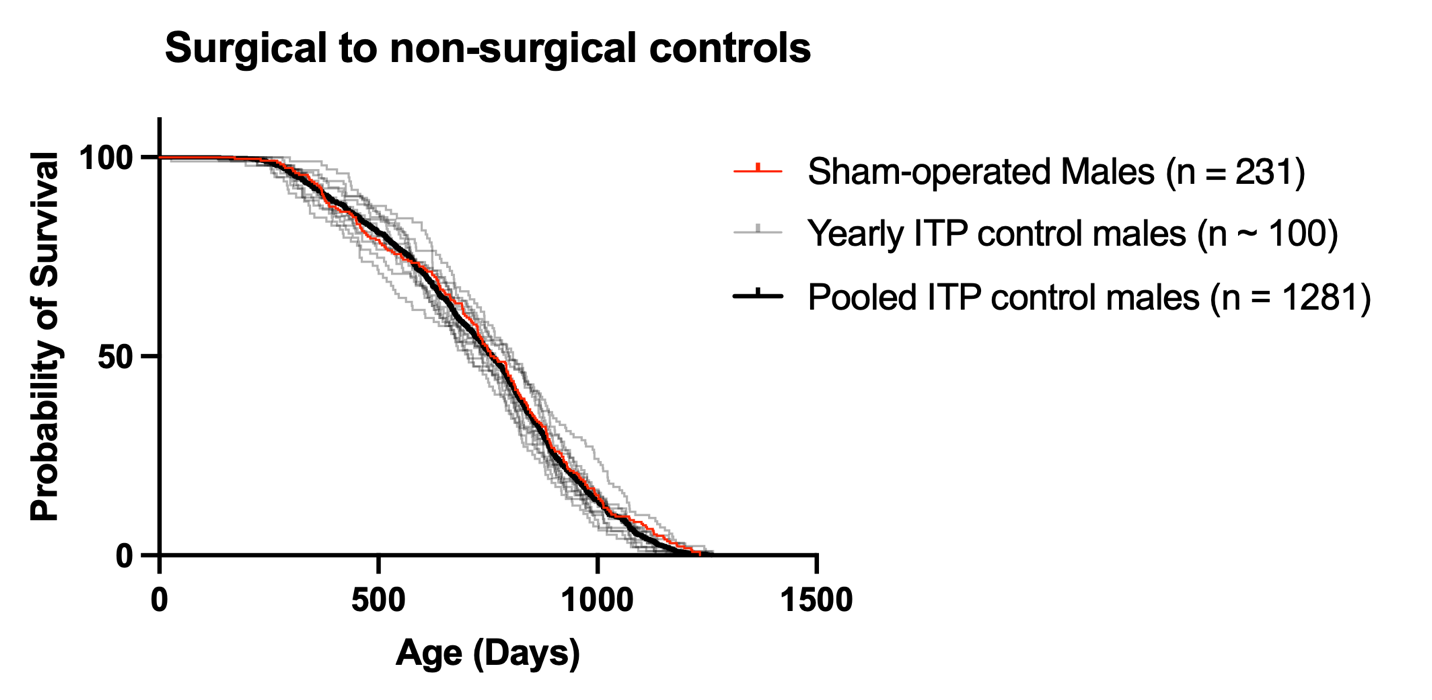


Figure S1, Survival curves for sham-operated control males and non-surgical controls from ITP.

Figure S2, Survival curves for SHAM males and minor wounded SHAM males.
